# Clinical Cases

**Published:** 1884-01

**Authors:** W. E. Leonard

**Affiliations:** Minneapolis, Minn.


### CLINICAL CASES.

W. E. LEONARD, M. D., MINNEAPOLIS.

CASE I.—November 15th, 1882.—Mrs. B., about forty, an actress, constantly using her voice, has had a severe sore throat for several days, almost incapacitating her for duty at the theatre.

At 4 P. M. I found both tonsils much swollen and inflamed, and presenting numerous depressions filled with yellowish mucus—a follicular tonsillitis; the fauces were red and painful. The right tonsil had been the first to be inflamed, but both were now alike. Some years before she passed through a severe attack of diphtheria. Her throat symptoms were much worse toward evening. Further inquiry elicited the fact that she had a very "weak stomach," having nearly lost her life from vomiting through the entire term of her last pregnancy—many years ago. Even now, with this complaint, she was much troubled with rumbling of gas in abdomen and frequent belching.

℞. Lycop.$^{cm}$, dose, and Sacc. lac. in water every two hours.

The next morning, before nine o'clock, she declared herself much better, having gone through "her part" the night before without pain.

On looking into the throat, the right tonsil was found reduced to quite its natural size, and less inflamed; the left had changed but little. She had used no other means for relief. A few powders of Sacc. lac. were left to be taken if she did not improve fast enough.

In a week she came to the office to say that she was entirely recovered, and had been on duty each night regularly.*

An epidemic of a like form of tonsillitis prevailed then throughout the city at the same time with diphtheria, being commonly diagnosed as "diphtheritic sore throat"—an improper mixing of terms, as I think.

*Lach.*, *Lyc.*, and *Merc. cyan.* cured such cases, according to my notes and the experience of others given at the time.

Case II.—February 23d, 1883.—Mrs. W., a typical blonde of large frame, lymphatic temperament, the mother of five children, menstruated about six months after her last confinement, and has now gone by one period since then without any flow.

Now, after being on her feet more than usual and being especially worried over household matters, she is suddenly seized with menorrhagia. At midnight she is found to have been flowing steadily for some hours, and is quite prostrated. Five times since noon had she fainted, once remaining unconscious fifteen minutes. The blood is hot, bright red, and comes in gushes, increasing on the least motion—she lies perfectly quiet on her back, and talks gaspingly.

She has never lost as much blood, even after confinements.

℞. *Bell.*$^{2c}$, in water every half hour until flow is checked, then hourly. Relief almost immediate, and no return.

---

* The only doubt of cure in my mind in this case was the fact that the *right* instead of the *left* tonsil improved first. According to the law of reverse order of symptoms, the left should have shown improvement first. Should it not?

